# Supplementary material for: Neuroinflammatory Response to TNFα and IL1β Cytokines Is Accompanied by an Increase in Glycolysis in Human Astrocytes In Vitro
Source: Int J Mol Sci. 2021 Apr 14;22(8):4065. doi: 10.3390/ijms22084065 (PMC8071021; doi:10.3390/ijms22084065)
Supplement: Supplementary file 1 [file ijms-22-04065-s001.zip › ijms-1167816-supp figures.docx]

Supplementary Figures


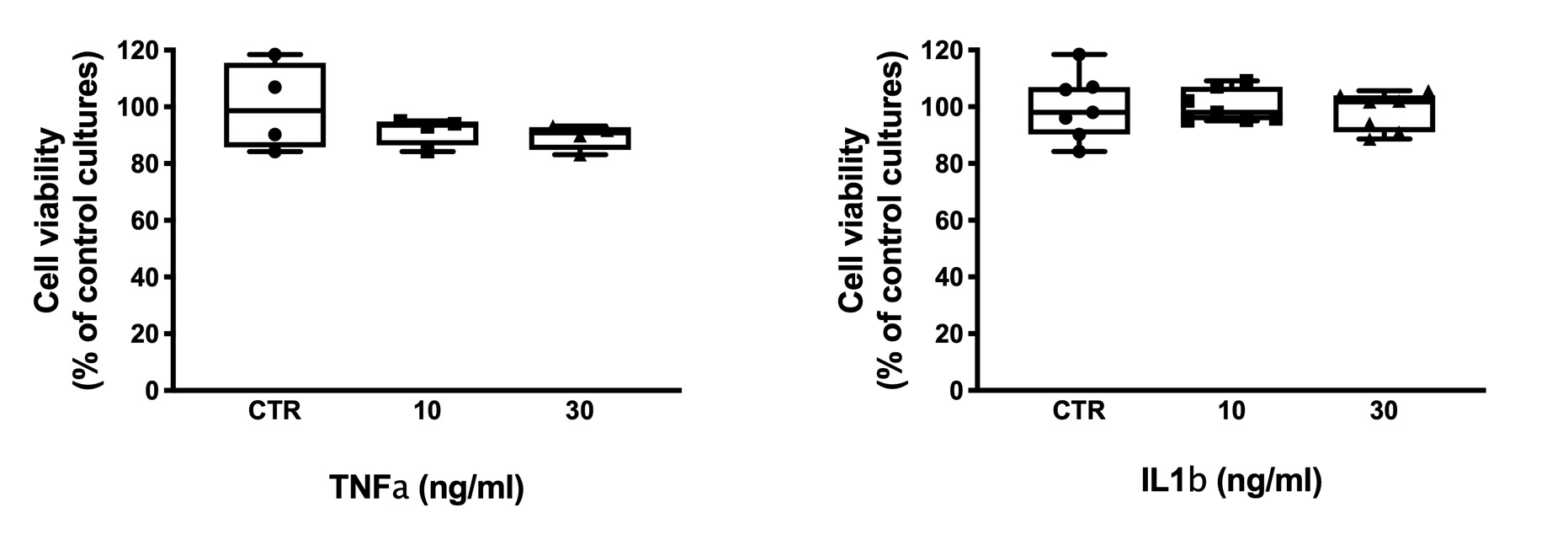


**Figure S1.** Absence of cytotoxicity after exposure to cytokines. Astrocytes viability was assessed by resazurin test after a 24h-exposure to three concentrations of TNFα or IL1β. Results are expressed as mean ± SD, for each group *n* = 7 samples coming from 2 independent experiments.


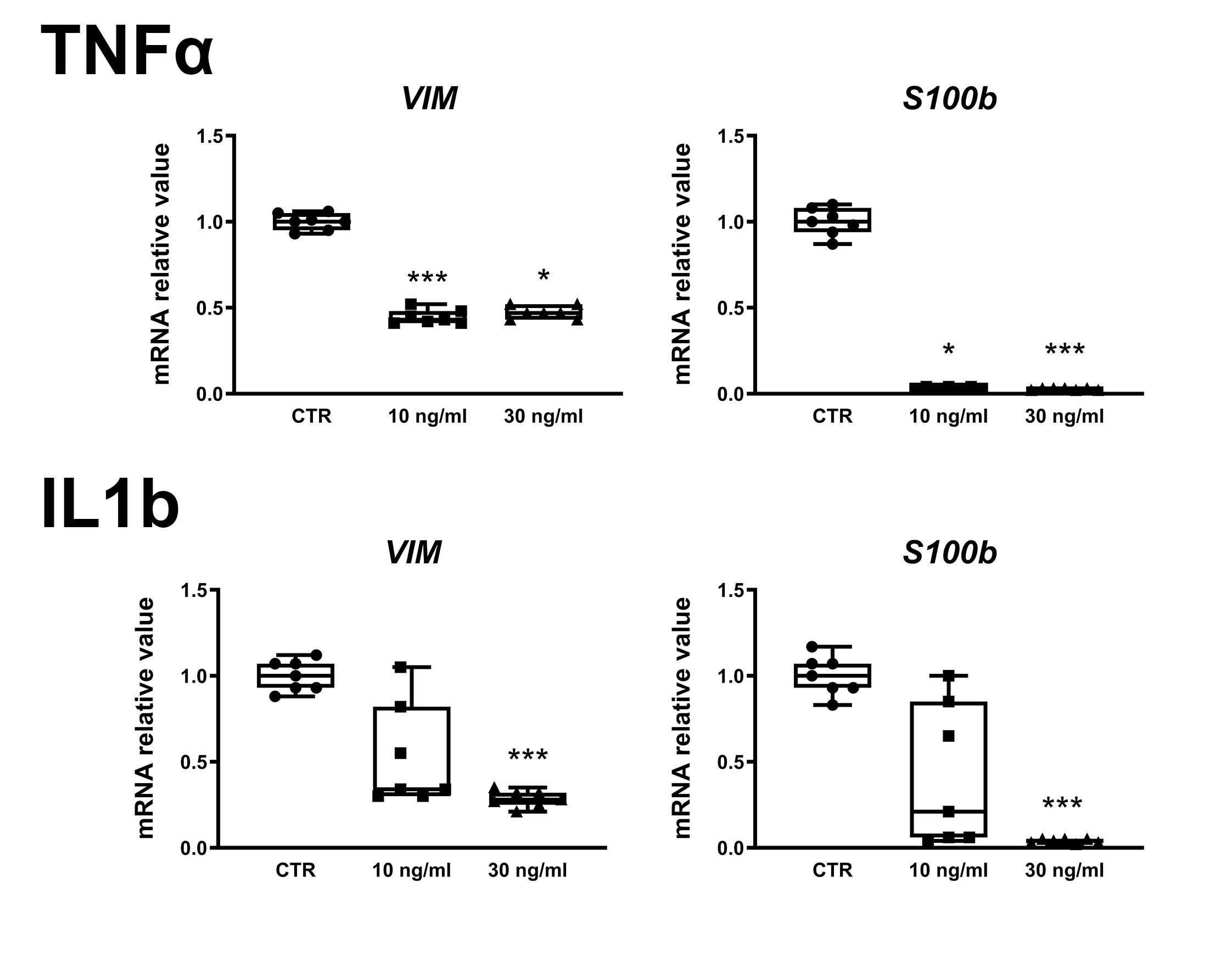


**Figure S2**. Exposure to cytokines downregulate vimentin and S100B gene expression in human ReN-derived astrocytes. Relative mRNA levels of astrocyte-specific genes after 24h-exposure to TNFα or to IL1β. Results are displayed as boxplots with data points, for each group *n* = 7 samples obtained in 2 independent experiments. The line in the box indicates the median, whereas top and bottom of the box represent the 75^th^ and 25^th^ percentiles; whiskers extend from minimum to maximum values. Statistical analysis was performed by using Kruskal-Wallis test followed by Dunn’s multiple comparisons test. **p* < 0.05; ****p* < 0.001.
