# Supplementary material for: Neuroinflammatory Response to TNFα and IL1β Cytokines Is Accompanied by an Increase in Glycolysis in Human Astrocytes In Vitro
Source: Int J Mol Sci. 2021 Apr 14;22(8):4065. doi: 10.3390/ijms22084065 (PMC8071021; doi:10.3390/ijms22084065)
Supplement: Supplementary file 1 [file ijms-22-04065-s001.zip › ijms-1167816-supp tables.docx]

Supplementary Tables

**Table S1.** List of primary and secondary antibodies used for immunohistochemistry.

| **Name** |  | **Species** | **Type** | **Ref** | **RRID** |
| --- | --- | --- | --- | --- | --- |
| GFAP | Primary | Anti-Rabbit | Polyclonal | Sigma  Cat# G9269 | RRID:AB_477035 |
| BTUIII | Primary | Anti-Rabbit | Monoclonal | Sigma  Cat# T8660 | RRID:AB_477590 |
| S100β | Primary | Anti-Rabbit | Polyclonal | Agilent  Cat#Z0311 | RRID:AB_10013383 |
| VIMENTIN | Primary | Anti-Mouse | Monoclonal | Abcam  Cat#ab8978 | RRID:AB_306907 |
| ALEXA 488 | Secondary | Donkey anti-mouse | Polyclonal | Thermo Fischer  Cat#A-11037 | RRID:AB_2534095 |
| ALEXA 594 | Secondary | Goat anti-rabbit | Polyclonal | Molecular Probes  Cat#A-21202 | RRID:AB_141607 |

**Table S2.** Sequences of qPCR primers used with SYBR green.

| **Gene** | **Forward primer (5’–3’)** | **Reverse primer (5’–3’)** |
| --- | --- | --- |
| *ACTB* | AAATCTGGCACCACACCTTC | AGAGGCGTACAGGGATAGCA |
| *B2M* | TGCTGTCTCCATGTTTGATGTATCT | TCTCTGCTCCCCACCTCTAAGT |
| *GFAP* | CACCACGATGTTCCTCTTGA | GTGCAGACCTTCTCCAACCT |
| *GLUT1* | GGCATTGATGACTCCAGTGTT | ATGGAGCCCAGCAGCAA |
| *GS* | TAAGGACCCTAACAAGCTGGT | CCGTTTACAGGTGTGCCTCAA |
| *IL1β* | TTCGACACATGGGATAACGAGG | TTCGACACATGGGATAACGAGG |
| *IL6* | CCTGAACCTTCCAAAGATGGC | TTCACCAGGCAAGTCTCCTCA |
| *Ki67* | GCCTGCTCGACCCTACAGA | GCTTGTCAACTGCGGTTGC |
| *MCT4* | CCATGCTCTACGGGACAGG | GCTTGCTGAAGTAGCGGTT |
| *PKM2* | GCCTGCTGTGTCGGAGAAG | CAGATGCCTTGCGGATGAATG |
| *PSMB8* | CACGCTCGCCTTCAAGTTC | AGGCACTAATGTAGGACCCAG |
| *TNFa* | GAGGCCAAGCCCTGGTATG | CGGGCCGATTGATCTCAGC |

**Table S3.** References of Taqman assays.

| **Gene** | **Assay ID (Thermofisher)** |
| --- | --- |
| *ACTB* | Hs01060665_g1 |
| *NFkB1* | Hs00765730_m1 |
| *NFkB2* | Hs01028890_g1 |
| *STAT1* | Hs01013996_m1 |
| *STAT3* | Hs00374280_m1 |
